# A genetic development route analysis on MDS subset carrying initial epigenetic gene mutations

**DOI:** 10.1038/s41598-019-55540-w

**Published:** 2020-01-21

**Authors:** Xiao Li, Feng Xu, Ling-Yun Wu, You-Shan Zhao, Juan Guo, Qi He, Zheng Zhang, Chun-Kang Chang, Dong Wu

**Affiliations:** Department of Hematology, Shanghai Jiao Tong University Affiliated Sixth People’s Hospital, Shanghai, China

**Keywords:** Cancer genetics, Myelodysplastic syndrome

## Abstract

MDS development is a dynamic process during which the accumulation of somatic mutations leads to specific malignant evolution. To elucidate the differential roles of gene mutations in typical MDS, we used targeted sequencing to investigate clonal patterns from 563 patients and focused on cases (199/563 cases) with initial mutations (ASXL1, DNMT3A and TET2) at MDS diagnosis. The consistency of frequency and distribution in patients with or without aberrant chromosomes suggested early events of these initial mutations. Some additional driver mutations (SF3B1, U2AF1 or RUNX1) played roles to keep the basic disease features, or give rise to different phenotypes (BCOR, EZH2 or TP53) in individual patients. Notably, analysis in paired samples before and after MDS progression showed that the mutations identified as last events (involving active signaling, myeloid transcription or tumor suppressor) seemed necessary for MDS development to be AML. Last mutations can exist at MDS diagnosis, or emerge at AML transformation, and involve a small group of genes. Single-allele CEBPA mutations and diverse TP53 mutations were checked as the most common last event mutations. Considering the necessity of last event mutations and limited gene involvement in AML transformations, it is possible to validate a small group of last events involved mutations to develop some new strategies to block MDS progression.

## Introduction

It is widely accepted that MDS-related mutations occur mainly in genes involved in mRNA splicing, epigenetic regulation, receptor and kinase signaling, transcription regulation, tumor suppression and adhesion^1–3^. Several in-depth studies have been completed to clarify the detailed mechanism underlying high-frequency mutations (such as SRSF2, ASXL1 and U2AF1)^4–6^. In addition, some hematologists are conducting longitudinal (over time) observation of evolutionary models of gene mutation in individual or groups of MDS patients to understand the onset and development of this disease^7–9^.

Jaiswal *et al*. reported clonal hematopoiesis at a frequency of approximately 10% in a large cohort of elderly individuals, presenting a low variant allele factor (VAF) of somatic mutations mainly in ASXL1, DNMT3A or TET2 but without any cytopenia^10^. A few of these cases transformed to MDS or AML subsequently. Other reports have proposed the retention of these initial mutations as a source of relapse after different therapies, including hypomethylation, chemotherapy, and even peripheral blood stem cell transplantation^7,9,11^. Apparently, the combinations of some new somatic (driver) mutations catalyze disease evolution and control disease phenotypes and outcomes. We hypothesize that MDS progression involves the following stages: the first stage only exhibits clone instability, and there is no hematopoiesis involvement (asymptomatic clonal hematopoiesis in aged populations). At the second stage, individuals exhibit not only unstable clones but also hematopoietic involvement without or with obvious changes in genetic material (typical MDS with normal or abnormal chromosomes). At the final stage, individuals develop AML by join of some last event mutation. In this study, we analyze and follow-up the characteristics of the additional driver gene mutation types (early drivers and last events) around the initial mutations (ASXL1.DNMT3A and TET2) at different MDS stages, seeking key gene mutations associated with phenotypic control and disease evolution of MDS.

## Results

### Patients

The data are from patients diagnosed in our department from July 2006 to June 2017. A total of 563 cases with a diagnosis of MDS (313 with normal karyotypes, 250 with abnormal chromosomes) were subjected to target sequencing (the general data for these cases can be found in Supplementary Table 1). Those with initial mutations were the focus of our study.

### Initial mutations

**Figure 1:**
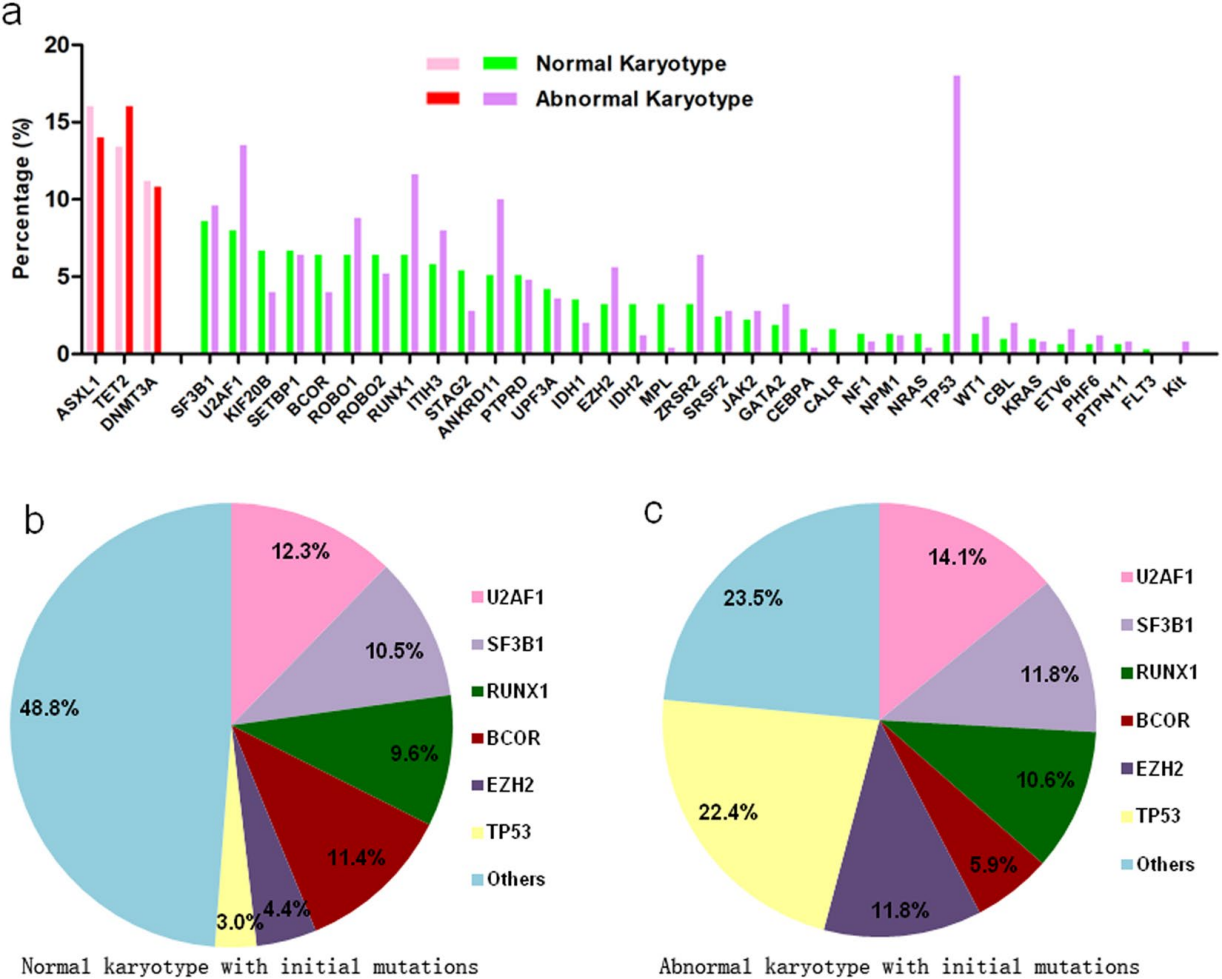
Distribution of initial and driver mutations. (**a**) Under the premise that the frequencies of the 3 initial mutations were very similar, the driver mutations were very different in patients with normal chromosomes or abnormal chromosomes (total 563 cases). Pink and red bar represents initial mutations (ASXL1, TET2 and DNMT3A) in patients groups with normal or abnormal karyotype, respectively. (**b**) Distribution of driver mutations in patients in C1 (114 cases, all with initial mutations). Driver mutations include U2AF1, SF3B1, RUNX1, BCOR, EZH2, TP53, etc. (**c**) Distribution of driver mutations in patients in C2 (85 cases, all with initial mutations). The frequencies of the U2AF1, SF3B1 and RUNX1 mutations were similar to those in C1, but that of BCOR mutations was lower, while that of EZH2/TP53 mutations was much higher.

Table 1 and Fig. 1a show the features of the initial mutations at the diagnosis of MDS. One hundred and fourteen (cohort 1, C1) out of the 313 cases (36.4%) with normal karyotypes carried one or two of the three initial somatic mutations. The frequencies of ASXL1, DNMT3A and TET2 mutations were 16.0%, 11.2% and 13.4%, respectively. Among these cases, a few exhibited dual initial mutations, while no individual exhibited triplex initial mutations. The mean VAF of the initial mutations from the 114 cases was 0.43 (range 0.11–0.88). Among the 50 cases with ASXL1 mutations, forty-two had mutations in exon 12. Thirteen of the 35 DNMT3A mutations were found in exon 23 (frequently reported as R882H/C). The TET2 mutation occurred mainly in exon 3 (25/42 cases) (Table 1). Simultaneously, eighty-five (cohort 2, C2) of the 250 cases (34.0%) with abnormal chromosomes carried one or two of the three initial somatic mutations. The frequencies of ASXL1, DNMT3A and TET2 mutations were 14.0%, 10.8% and 16.0%, respectively. The other features of these cases can be found in Table 1 and were very similar to those of cohort1.

**Table 1:** Comparison of initial mutations for all 563 cases.

Parameters	Normal chromo	Abnormal chromo	*P*
Initial mutation number	114/313 cases	85/250 cases	
Initial mutation (%)	36.4	34.0	0.584
ASXL1 (%) (No) (exon12)	16.0(50) (42/50)	14.0(35)(31/35)	0.878
DNMT3A (%) (No) (exon23)	11.2(35) (13/35)	10.8(27)(10/27)	0.995
TET2 (%) (No) (exon3)	13.4(42) (25/42)	16.0(40)(21/40)	0.734
Mean VAF (range)	0.43(0.11–0.88)	0.45(0.23–0.86)	
ASXL1 + DNMT3A (no)	3	2	
ASXL1 + TET2 (no)	6	10	
DNMT3A + TET2(no)	5	5	
ASXL1 + DNMT3A + TET2 (no)	0	0

### Baseline additional driver mutations

Coexisting additional driver mutations at diagnosis were found in 89 (78.1%) out of the 114 cases in C1. Accordingly, the coexisting mutations with frequencies of or greater than approximately 10% were U2AF1 (12.3%), BCOR (11.4%), SF3B1 (10.5%) and RUNX1 (9.6%) (Table 2 and Fig. 1b). Coexisting additional driver mutations were found in 69 (81.2%) of the 85 cases in C2. Accordingly, the coexisting mutations with frequencies of or greater than approximately 10% were TP53 (22.4%), U2AF1 (14.1%), EZH2 (11.8%), SF3B1 (11.8%), and RUNX1 (10.6%) (Table 2 and Fig. 1c). In general, the frequencies of SF3B1 and U2AF1 (RNA-splicing-related genes) as well as RUNX1 (myeloid transcription factor) were high and frequently similar in C1 and C2. In addition to the three high-ratio mutations, BCOR was the only high-ratio driver mutation in C1, whereas TP53 and EZH2 (especially TP53) constituted the fundamental fraction of the driver mutations in C2.

**Table 2:** Driver mutations for initial mutation positive case.

parameters	Normal chromosome	Abnormal chromosome	*P*
Driver mutation(%)	78.1(89/114 cases)	81.2(69/85 cases)	0.592
TP53 mutation (%)	2.6	22.4	<***0***.***001***
EZH2 mutation (%)	4.4	11.8	***0***.***051***
BCOR mutation (%)	11.4	5.9	*0*.*179*
U2AF1 mutation (%)	12.3	14.1	0.704
SF3B1 mutation (%)	10.5	11.8	0.783
RUNX1 mutation (%)	9.6	10.6	0.631

Note: The genes listed here are those their mutation rate ≥10% at least in one subset.

### Phenotypes and outcome

**Figure 2:**
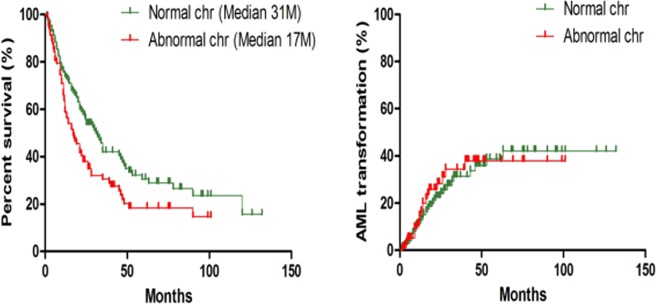
Survival and AML transformations for C1 and C2. Ptients with abnormal chromosomes exhibited very short survival times.

The clinical features of C1 are shown in Table 3. In general, these cases were predominantly classified as having low International Prognostic Scoring System (IPSS) scores (82.5%, 94 cases with ≤1.0, including 44.7% with refractory cytopenia with multilineage dysplasia [RCMD] and 34.2% with refractory anemia with excess blasts [RAEB]. The determinant AML transformation occurred in 26.3% of the patients (30 out of the 114 cases), and the median survival of the 114 patients was 47 months (Fig. 2). The phenotypes of C2 are also shown in Table 3. Compared with the patients with normal chromosomes, the patients in this group appeared to be advanced cases (IPSS ≥1.5, RAEB and higher blast proportion). During the same follow-up period, the determinant AML transformation occurred in 24.7% of the patients (21 out of the 85 cases), and the median survival of the 85 patients was only 17 months (Fig. 2).

**Table 3:** Comparison of clinical features.

Parameters	Normal chromo	Abnormal chromo	*P*
Cases number	114	85	
Median age (years)	60	62	0.075
Sex (male: female)	1.78	1.66	**0**.**808**
IPSS scoring ≤1.0 (%)	82.5	47.1	**<*0***.***001***
RCMD (%)	44.7	29.4	*0*.*028*
RAEB1 + 2(%)	34.2	41.2	0.315
**Chromosome (%)**			
Complex		26/85 (30.6)	
Trisomy 8		15/85 (17.6)	
20q-		11/85 (12.9)	
7q-/-7		9/85 (10.6)	
5q-/-5		10/85 (11.8)	
The others		20/85 (23.5)	
Cellulerity (median) (%)	60	70	0.124
BM Blast Ratio(median) (%)	2.4	3.6	0.610
Hb (g/L) (median)	73	72	0.426
WBC (×10^9^/L) (median)	3.0	3.7	0.155
Neutrophil (%)	49	50	0.352
BPC (×10^9^/L) (median)	47	50	0.211
AML transformation (%)	26.3	24.7	0.797
Total median survival (M)	31	17	***0***.***016***

### Gene characteristics around the AML transformation

**Figure 3:**
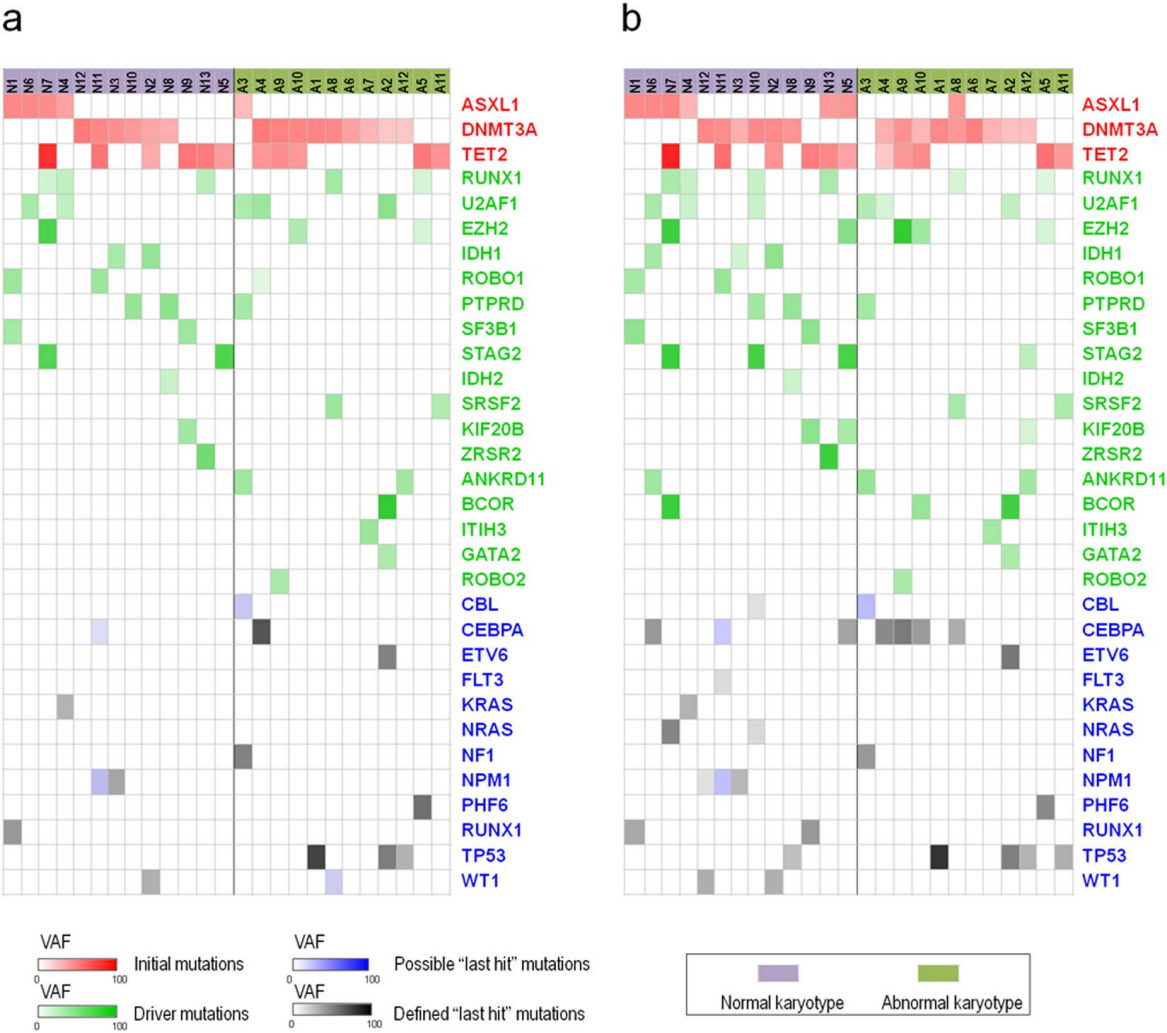
Evolution of initial/driver and last event mutations in 25 paired samples pre and post AML transformation. (**a**) Distribution of initial/driver and last event mutations for 25 cases at MDS diagnosis. The blue panel represents the mutations that met the criteria for last events; the black panel represents the defined last events mutations according to the criteria described in the text. (**b**) Distribution of initial/driver and last event mutations for 25 cases after AML transformation. The blue and black panels represent the same mutations described above.

**Figure 4:**
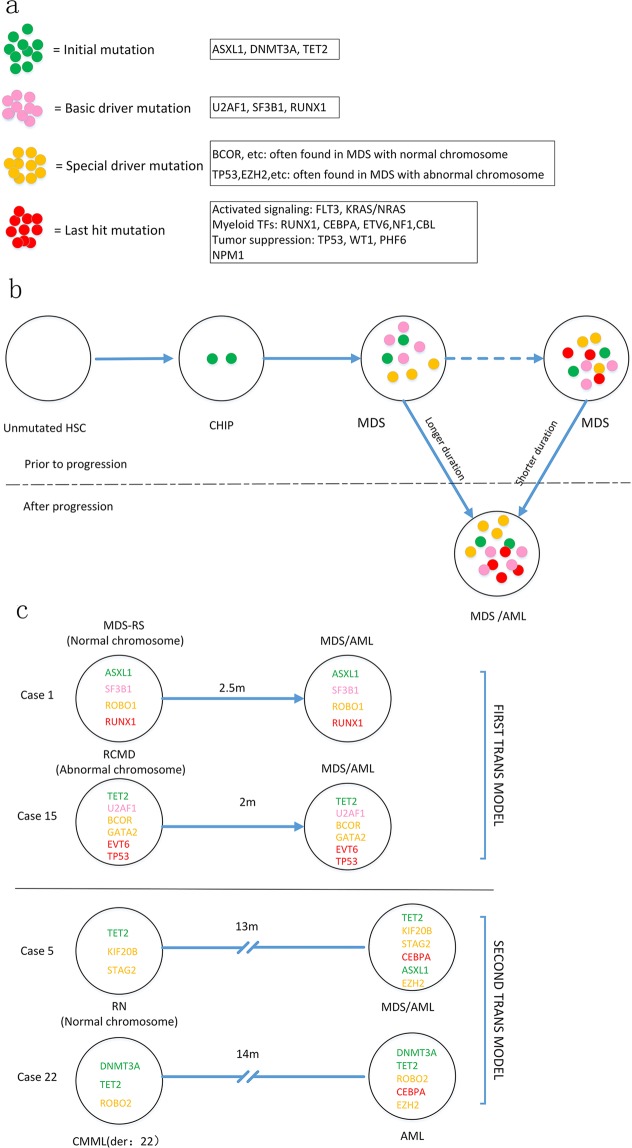
Schematic depiction of MDS evolution. (**a**) Schematic diagram of different mutations. (**b**) Generalized pattern of clonal evolution summarized from this study. C: Illustration of individual MDS/AML. Font color in cells represents the properties of the mutations.

A total of 30 patients in C1 and 21 patients in C2 exhibited transformation to AML upon follow-up. To determine whether each patient who underwent AML transformation needed a last mutation events, we performed target sequencing of 25 cases from C1/C2 before (at diagnosis of MDS) and after (immediately after MDS/AML diagnosis) AML transformation (Fig. 3). For gene mutations to be defined as last events, they needed to meet one of the following criteria: 1. there was no emerged mutations after MDS/AML diagnosis, but at least one of the baseline gene mutations involved one of following pathway: activated signaling, myeloid transcriptional factors, tumor suppressors or NPM1, and the AML occurred in less than one year from MDS diagnosis (transformation style1 in Fig. 4). 2. the patients were defined with newly emerged gene mutations immediately after the MDS/AML diagnosis, and at least one of the emerged mutation involved the four pathway mentioned above(transformation style2 in Fig. 4). Whereas for those who transformed to be AML but lacked post-transformation samples, the existed mutations (mentioned above) at MDS diagnosis could not considered as last events, because some more aggressive mutations could emerge during AML transformation (such as in case number 11, as shown in Supplementary Table 3). Supplementary Tables 2, 3 and Fig. 3 show that twenty-two of the 25 patients exhibited evidence of last mutation events. Supplementary Tables 2 and 3 show that AML transformation style 1 requires a shorter duration than style 2: 5.9 and 5.4 months in C1 and C2, respectively. While the secondary transformation style requires relatively long durations (16.8 and 8 months for C1 and C2, respectively), In general, those with abnormal chromosomes exhibited relatively short AML transformation times (6.7 months for 10 patients in C2 vs. 14 months for 12 patients in C1). Overall, the last events involved a total of eleven genes: CBL, CEBPA, ETV6, FLT3, NF1, NPM1, PHF6, KRAS/NRAS, TP53, RUNX1 and WT1. High-frequency mutations in CEBPA (6 cases) and TP53 (5 cases) are highlighted.

## Discussion

The description of initial (founder) gene mutations before and during MDS development has been widely accepted^12^. Mutations such as ASXL1, DNMT3A and TET2 are widely considered to be the most typical initial mutations^9,10^. Mossner *et al*. transferred CD34+ cells (containing several VAF closed gene mutations, including ASXL1) and MSCs from MDS patients into mice with a pure background^9^. After 14 weeks, the human original hematopoietic cells in mice could be checked only with ASXL1 mutations, which were then deduced as the initial mutations that lead to MDS development. In general, initial mutations were considered the founder factors that emerged before the clinical phenotypes of MDS^10,13,14^. Typical MDS symptoms appeared over time or upon the introduction of new driver gene mutations. When the additional gene mutations involve activation signals, myeloid transcriptional factors, or tumor suppressors, MDS may transform into AML^15,16^. Hypomethylation, chemotherapy, and even PBSCT could clear the driver mutations, leading to complete remission (CR), but the initial mutation in the bone marrow was retained as a residue of the disease and was the source of disease relapse^9,11^. The clinical phenotype could completely change if the founder is combined with different driver mutations upon disease relapse. Apparently, the initial mutations lead to the origination and relapse of MDS. Whereas, the additional somatic mutations (drivers) give rise to the phenotypes of MDS. Finally, the combinations of some specific mutations as last events lead to the AML transformation. We hope to primarily prove the process by longitudinal target sequencing for those MDS subsets who had with the admitted initial mutations at diagnosis.

One hundred and ninety-nine (35.1%) out of the 563 MDS cases diagnosed in our department were defined by at least one of the three initial mutations by sequencing 38 target genes. There were two pieces of evidence to support the idea that the initial mutations are early events in MDS development. First, thirty-one (15.6%) out of the 199 cases in this assay were defined by two of the three initial mutations, but none of these cases had all three initial mutations. Considering the predicted roles that the three genes play in epigenetic regulation, one or two initial mutations appear to be sufficient for successful MDS origination. In addition, the patients in C1 and C2 presented totally different phenotypes in this assay, whereas the characteristics of these patients in terms of initial mutations appeared to be very consistent, regardless of frequency, location and VAF, strongly indicating that the initial mutations are early events during MDS development (Table 1 and Fig. 1a).

After the consistent features of the initial mutations were defined in C1 and C2, we asked why the patients in C2 exhibited completely different clinical phenotypes with increased age, increased risk ratios, rapid AML transformation and decreased survival (Table 3 and Fig. 2). We focused on only driver mutations. First, we observed that the mutation frequencies in SF3B1 and U2AF1 were very similar between the two cohorts (12.3% vs 14.1% and 10.5% vs 11.8%, respectively), which seemed reasonable. Considering the important roles that RNA splicing plays in MDS pathogenesis, mutations in the most common RNA splicing regulators should be indispensable during MDS development^5,6,17,18^. On one hand, these mutations lead to MDS-specific phenotypes (such as mutations in SF3B1, which lead increased levels of ring sideroblasts)^18^. On the other hand, these mutations may lead to some other MDS-specific features, such as overapoptosis or dysplasia^4,19^. In addition to SF3B1 and U2AF1, the RUNX1 mutation ratio was very similar in C1 and C2 (9.6% vs 10.6%). As a myeloid transcription factor, RUNX1 should be essential for the maintenance of MDS features and progression regardless of chromosomal status^20,21^. TP53 (22.4%) and EZH2 (11.8%) were the most predominant mutations in C2. Understandably, the high frequency of TP53 mutations (causing genetic instability) and EZH2 mutations (located on chromosome 7 and often coexisting with chromosome 7 abnormalities) led to poor prognoses for patients in C2^22,23^. Apparently, the differentiated driver mutation styles resulted in different phenotypes and outcomes for individual patients under the premise of the same initial mutations.

Sequencing results were analyzed from patients who exhibited transformation from MDS to AML. We focused on the last mutation events from 25 patients whose paired samples (before and after AML transformations) were successfully acquired. The last events were systematically defined (described in Results part) to rule out perturbations. Twenty-two of the 25 available patients were defined with last events mutations, with a high frequency of involvement in CEBPA and TP53 but none in C-Kit. Considering the limitations of target sequencing, we speculate that each MDS/AML case may need last events mutations for AML transformation. In addition, considering that M2 is a common subset of MDS/AML, it is acceptable that CEBPA is a highly frequent last events, although there have been no similar reports.

## Conclusions

The last event mutations may be necessary in AML transformations. Further validation should be carried out to verify our results, and if the last mutations are truly limited within a small spectrum (as observed in this assay), these mutations may represent a new targeted or immune therapy to block MDS progression.

## Methods

### Patients and samples

588 samples from 563 MDS patients from 2006 to 2017 were involved into this study. MDS diagnosis meets the minimum diagnostic criteria (Vienna, 2006)^24^. MDS classification and prognosis were evaluated according to the WHO criteria and the International Prognostic Scoring System (IPSS)^25,26^. Bone marrow mononuclear cells were obtained by density gradient centrifugation. All of the subjects provided written informed consent for genetic analysis under a protocol that was approved by the Ethics Committee of Shanghai Jiao Tong University Affiliated Sixth People’s Hospital. All methods were performed in accordance with the relevant guidelines and regulations.

### Genomic DNA preparation

Genomic DNA (gDNA) was extracted from bone marrow mononuclear cells. The purity (OD260/280 > 1.8) and concentration (50 ng per µl) of the gDNA met the sequencing requirements. Sequencing methods and statistical analysis are referred in our previous study^27^.

### Targeted gene sequencing

Thirty-eight genes (ASXL1, ANKRD11, BCOR, CALR, CBL, CEBPA, DNMT3A, ETV6, EZH2, FLT3, GATA2, IDH1, IDH2, ITIH3, JAK2, KIF20B, KIT, KRAS, MPL, NF1, NPM1, NRAS, PHF6, PTPN11, PTPRD, ROBO1, ROBO2, RUNX1, SETBP1, SF3B1, SRSF2, STAG2, TET2, TP53, U2AF1, UPF3A, WT1 and ZRSR2)^2,27^ were examined for mutations by MiSeq sequencing (Illumina, San Diego, CA, USA). To identify mutations in the selected genes, we designed PCR primers using the primerXL pipeline. A total of 755 oligonucleotide pairs were produced, encompassing all of the coding sequences (CDSs) and most of the UTRs of the 38 genes. The amplification reactions were conducted using an ABI 2720 thermal cycler. The PCR products were used to generate a library for further detection, and the adapter-ligated and adapter-indexed DNA fragments from 10 libraries were then pooled and hybridized. After hybridization of the sequencing primer, base incorporation was performed in a single lane using a MiSeq benchtop sequencer, following the manufacturer’s standard cluster generation and sequencing protocols, for 250 cycles of sequencing per read to generate paired-end reads that included 250 bp at each end and 8 bp of the index tag.

### Mutation calling

Sequence data were analyzed via our established pipeline to detect possible somatic mutations. All of the sequencing reads were aligned to the human reference genome (hg19) using BWA version 0.5.8 with default parameters. After all of the duplicated reads and low-quality reads and bases were removed, the allele variable frequencies (AVFs) of single-nucleotide variants (SNVs) and indels were calculated at each genomic position by enumerating the relevant reads using SAMtools. All of the variants that exhibited AVF > 10% were extracted and annotated with ANNOVAR for further consideration only if they were identified in >5 positive reads among >10 total reads. All of the synonymous variants and ambiguous (unknown) candidates were discarded. In addition, known SNVs with frequencies greater than 0.01 in the 1000 Genomes databases were removed, although SNVs that were found in the COSMIC database were rescued as somatic mutations.

### Statistical analysis

Statistical analyses were conducted using SPSS software version 18.0. The associations of the mutations with clinical characteristics were analyzed via the χ^2^ test. Comparisons of two independent samples were assessed using the two-tailed Student’s t-test. Fisher’s exact test was applied to determine the co-occurrence of highly recurrent genes. Kaplan-Meier analysis was used to evaluate the time to survival and time to progression. All *P*-values were based on 2-sided tests and *P*-values less than 0.05 were considered statistically significant.

## Supplementary information

Supplementary Tables

## Data Availability

The sequencing data can be obtained by contacting the corresponding author.
