# Supplementary material for: A genetic development route analysis on MDS subset carrying initial epigenetic gene mutations
Source: Sci Rep. 2020 Jan 21;10:826. doi: 10.1038/s41598-019-55540-w (PMC6972820; doi:10.1038/s41598-019-55540-w)
Supplement: Supplementary file 1 — Supplementary Tables [file 41598_2019_55540_MOESM1_ESM.doc]

**A genetic development route analysis on MDS subset carrying initial epigenetic gene mutations**

Xiao Li^1,2,*^, Feng Xu^1,2^, Ling-Yun Wu^1,2^, You-Shan Zhao^1^, Juan Guo^1^, Qi He^1^, Zheng Zhang^1^, Chun-Kang Chang^1^, Dong Wu^1^

^1^Department of Hematology, Shanghai Jiao Tong University Affiliated Sixth People's Hospital

^2^These authors contributed equally to this work

Running Title: Mutation evolution in MDS

^*^Correspondence to: Xiao Li, MD, PhD, Dept. of Hematology, Shanghai JiaoTong University Affiliated Sixth People’s Hospital, Shanghai, 200233, China.

Email: [lixiao3326@163.com](mailto:lixiao3326@yahoo.com.cn), Tel.: +86-021-24058745, Fax: +86-021-64701361

This study was supported by the National Natural Science Foundation of China (grant nos. 81470291, 81770120 and 81770122) and Shanghai Health Commission General Program (grant nos.201640267).

**Supplementary Table1. Features of tested patients**

| **Parameters** | **Normal chromosome** | **Abnormal chromosome** |
| --- | --- | --- |
| **Cases number** | 313 | 250 |
| **Median age (years)** | 56 | 60 |
| **Sex (male: female)** | 1.30（177:136） | 1.58(153:97) |
| **IPSS scoring ≤1.0 (%)** | 88.2 （276/313 cases）） | 59.2(148/250 cases) |
| **IPSS scoring ≥1.5(%)** | 11.8 （37/313cases） | 40.8 (102/250 cases) |
| **Mut Frequency (%)** | 80.2 | 89.1 |
| **Mut No/per cases (median /mean) (range)** | 2.0/2.0 (0-7) | 2.0/2.1 (0-7) |
| **Fre of initial Mut (%)** | **36.4** | **34.0** |

**Supplementary Table 2 Characteristics of the last events for patients with normal chromosome**

| **Patient** | **ID** | **Mut No** | **Karyotype** | **Diagnosis** | **IPSS (R)** | **AML Trans (M)** | **Survival**  **(M)** | **Gene Mutations** |
| --- | --- | --- | --- | --- | --- | --- | --- | --- |
| **Last events existed during MDS stage (mean duration for AML transformation was 5.9 months)** | | | | | | | | |
| 1. F/59 | 3636 | **4** | normal | RCMD-RS | 0.5（2.5） | **2.5** | **5** | ASXL1/12/L1266V（47）  ROBO1/4:c.89-5G>A（48）  **RUNX1/4/H105Y（40）**  SF3B1/15/K700E（45） |
|  | 3651 | **4** | del(5)q(31)[14] | AML |  |  |  | ASXL1/12/L1266V（47）  ROBO1/4:c.89-5G>A（44）  **RUNX1/4/H105Y（34）**  SF3B1/15/K700E（55） |
| 2. M/60 | 1849 | **4** | normal | RCMD | 0.5(2.0) | **8** | **25** | DNMT3A/23/R882C（35）  IDH1/4/I99M（52）  TET2/3/H702fs（33）  **WT1/7/H428fs（31）** |
|  | 2151 | **4** | normal | AML |  |  |  | DNMT3A/23/R882C（35）  IDH1/4/I99M（52）  TET2/3/H702fs（33）  **WT1/7/H428fs（31）** |
| 3. F/47 | 2036 | **3** | normal | RAEB2 | 2.0（5.5）） | **11** | **14.5** | DNMt3A/23/R882H(44)  IDH1/4/R132H（43）  **NPM1/11/L287f（35）** |
|  | 2420 | **3** | normal | AML |  |  |  | DNMt3A/23/R882H(31)  IDH1/4/R132H（23）  **NPM1/11/L287f（28）** |
| 4. M/33 | 2998 | **4** | normal | RAEB2 | 2.0（6.5）） | **2** | **11** | ASXL1/12/R693X(37)  **KRAS/2/G12S(30)**  RUNX1/9/967+1>A6/886+1>A(31)  U2AF1/2/S34F(31) |
|  | 3080 | **4** | normal | AML |  |  |  | ASXL1/12/R693X(37)  **KRAS/2/G12S(30)**  RUNX1/9/967+1>A6/886+1>A(31)  U2AF1/2/S34F(31) |
| **Last events emerged immediately after MDS/AML diagnosis(mean duration for AML transformation was 16.8 months)** | | | | | | | | |
| 5. M/60 | 3288 | **4** | normal | RN | 0.0（2.0） | **13** | **13** | KIF20B/20/T1175N(48)  STAG2/7/S189fs（88）  TET2/3/p612-610del(50)/6/P1239del(47) |
|  | 3741 | **7** | normal | AML |  |  |  | KIF20B/20/T1175N(43)  STAG2/7/S189fs（89）  TET2/3/p612-610del(39)/6/P1239del(36)  ASXL1/12/G642fs(41**)**  **CEBPA/1/L338P(36)**  EZH2/20/R746G(59) |
| 6. M/70 | 1243 | **2** | normal | RCMD | 0.5(4.0) | **18** | **22** | ASXL1/12/G642fs  U2AF1/2/S34F |
|  | 1925 | **5** | normal | AML |  |  |  | ASXL1/12/G642fs(47)  U2AF1/2/S34F(43)  ANKRD11/6/S128F（47）  **CEBPA/1/7_15del（40）**IDH1/4/R132C（44） |
| 7. M/62 | 809 | **7** | normal | RCMD | 0.5(3.5) | **17** | **20** | ASXL1/12/R715fs（45）  EZH2/18/1948-1G>T（88）  RUNX1/6/508+1G>C(24)/5/G170R(23)  STAG2/16/W485(84)  TET2/6/R1262P(81) |
|  | 1406 | **8** | normal | AML |  |  |  | ASXL1/12/R715fs (48)  EZH2/18/1948-1G>T(95)  RUNX1/6/508+1G>C(45)/5/G170R(42)  STAG2/16/W485(96)  TET2/6/R1262P(87)  BCOR/14/G1625E(96)  **NRAS/2G13R(48)** |
| 8. M/59 | 2666 | **3** | normal | RAEB1 | 0.5（4.0） | **10** | **37** | DNMT3A/19/K766fs(32)  IDH2/4/R140Q(27)  PTPRD/27/G809V(56); |
|  | 3020 | **4** | normal | AML |  |  |  | DNMT3A/19/K766fs(42)  IDH2/4/R140Q(25)  PTPRD/27/G809V(51);  **TP53/5/P151S(25)** |
| 9. M/59 | 1582 | **3** | normal | CMML1-RS | 0.0(2.5) | **14** | **17** | KIF20B/17/N716D(46)  SF3B1/14/K666T(48)  TET2/3/S1039L(54) |
|  | 2076 | **4** | normal | AML |  |  |  | KIF20B/17/N716D(56)  SF3B1/14/K666T(56)  TET2/3/S1039L(51)  **RUNX1/9/P340fs(40)** |
| 10.M/67 | 1057 | **2** | normal | RAEB1 | 0.5（4.5）） | **48** | **53** | DNMt3A/14/L547H（40）  PTPRD/10/A539V（51） |
|  | 2971 | **6** | normal | AML |  |  |  | DNMt3A/14/L547H（46）  PTPRD/10/A539V（46）  **CBL/8/C384R(12)**  **NRAS/2/3/G12V/Q61L(15)**  RUNX1/4/L98fs(28)  U2AF1/2/S34F(26) |
| 11.F/65 | 2577 | **5** | normal | CMML1 | 1.0（5.5）） | **11** | **14** | CEBPA/1/p69-70del(17)  DNMT3A/23/R882C(47)  NPM1/10/L258fs(33)  ROBO1/24/L1171F(49)  TET2/6/p1217-1222del(54) |
|  | 2951 | **6** | normal | AML |  |  |  | CEBPA/1/p69-70del(21)  DNMT3A/23/R882C(45)  **FLT3/14/c.1804_1805insGAGAATATGAATATGATCTCA:p.K602delinsREYEYDLK(14)**  NPM1/10/L258fs(25)  ROBO1/24/L1171F(50)  TET2/6/p1217-1222del(57) |
| 12.M/61 | 3567 | **1** | normal | RAEB2 | 1.5（4.5）） | **3** | **4** | DNMT3A/18/T645A(52) |
|  | 3684 | **3** | normal | AML |  |  |  | DNMT3A/18/T645A(47)  **NPM1/10/10/W259delineWQ(25)R262fs(12)**  **WT1/7/S169fs(32)** |
| **Newly emerged mutations not meet the criteria of last events** | | | | | | | | |
| ★13.M/61 | 3479 | **3** | normal | RCMD | 0.5（1.5） | **42** | **45** | RUNX1/3/R174Q(35)  TET2/11/Y1649S(51)  ZRSR2/7/R169X(69) |
|  | 4043 | **5** | normal | AML |  |  |  | ASXL1/11/P519fs(39)  RUNX1/3/R174Q(42)  TET2/11/Y1649S(44)  TET2/3/I1116fs(47)  ZRSR2/7/R169X(95) |

**Note:** boldfaced characters in the “gene mutation” column presented the presumptive last hits mutations met the criteria described in text

**Supplementary Table 3 characteristics of the last events for patients with abnormal chromosome**

| **Patient** | **ID** | **Mut No** | **Chromosome** | **Diagnosis** | **IPSS（R）** | **AML tran（m)** | **Survival**  **（m）** | **Gene Mutations** |
| --- | --- | --- | --- | --- | --- | --- | --- | --- |
| **Last events existed during MDS stage (mean duration for AML was 5.4 months)** | | | | | | | | |
| 1. F/71 | 1694 | **2** | complex | RCMD | 1.5(6.0) | **12** | **12** | DNMT3A/23:/R882C(48)  **TP53/7/M237I(74)** |
|  | 2131 | **2** | complex | AML |  |  |  | DNMT3A/23:/R882C(47)  **TP53/7/M237I(80)** |
| 2. M/58 | 1670 | **5** | 46,XY,del(18) | RCMD | 1.0(5.5) | **2** | **9** | BCOR/9/3745+7G>A(100)  **ETV6/5/V285M(49)**  GATA2/5/M388fs(41)  **TP53/2/E11Q(51)**  U2AF1/2/S34Y（57） |
|  | 1735 | **5** | The same | AML |  |  |  | BCOR/9/3745+7G>A(93)  **ETV6/5/V285M(53)**  GATA2/5/M388fs(41)  **TP53/2/E11Q(50)**  U2AF1/2/S34Y（30） |
| 3. F/40 | 2206 | **5** | 47<XX,+8] | RCMD | 1.0(5.0) | **3** | **16** | ANKRD11/11/.R2506K(47)  CBL/8/C396R（27）  **NF1/17/M645V(49)**  PTPRD/29A1047S(44)  U2AF1/2/S34Y(40) |
|  | 2301 | **5** | The same | AML |  |  |  | ANKRD11/11/.R2506K(52)  CBL/8/C396R（27）  **NF1/17/M645V(40)**  PTPRD/29A1047S(45)  U2AF1/2/S34Y(38) |
| 4.M/78 | 1931 | **4** | complex | RAEB1 | 2.0（7.0）） | **8** | **9** | **CEBPA/1/S9F(67)**  DNMT3A/12/1429+2T>A(52)  TET2/3/H682fs(41)  U2AF1/2/S34F(49) |
|  | 2221 | **4** | complex | AML |  |  |  | **CEBPA/1/S9F(46)**  DNMT3A/12/1429+2T>A(32)  TET2/3/H682fs(22  U2AF1/2/S34F(21) |
| 5.F/42 | 1350 | **4** | Complex | RAEB1 | 2.0（8.0） | **2** | **17** | EZH2/12/R497Q(21)  **PHF6/9/Y303X(56)**  RUNX1/9/P333fs(20)  TET2/11/Y1631H(53) |
|  | 1417 | **4** | Complex | AML |  |  |  | EZH2/12/R497Q(21)  **PHF6/9/Y303X(46)**  RUNX1/9/P333fs(17)  TET2/11/Y1631H(56) |
| **No mutations meet the criteria of last events pro and after MDS/AML diagnosis** | | | | | | | | |
| *6.M/63 | 3437 | **1** | 46,XY,inv(9)(p21q21) | RA | 0.5（3.5） | **3** | **12** | DNMT3A/16/1668-2A>G(37) |
|  | 3539 | **1** |  | AML |  |  |  | DNMT3A/11/c1212-2A>G(50) |
| *7.F/62 | 1619 | **3** | complex | RAEB2 | 3.0(8.5) | **24** | **28** | DNMT3A/23/R882C  ITIH3/5/20/V173F(75)/V777I（50） |
|  | 2589 | **3** | complex | AML |  |  |  | DNMT3A/23/R882C(30)  ITIH3/5/20/V173F(54)/V777I（48） |
| **Last events emerged immediately after MDS/AML(mean duration for AML transformation was 8.0 months)** | | | | | | | | |
| 8.M/77 | 1090 | **3** | 46，XY，del(20） | RARS | 0.0（3.5） | **9** | **11** | DNMt3A/23/R882H（47）  RUNX1/6/D317G（45）  SRSF2/1/P95R（49） |
|  | 1511 | **6** | Same | AML |  |  |  | DNMt3A/23/R882H（41）  RUNX1/6/D317G（48）  SRSF2/1/P95R（43）  ASXL1/12/P808fs(40)  **CEBPA/1/Y285fs(32)**  SETBP1/4/D868N(16) |
| 9. M/53 | 1891 | **3** | 46,XY，der(22) | CMML1 | 1.0（4.5）） | **14** | **18** | DNMt3A/23/R882C(49)  ROBO2/13/R640H(42)  TET2/10/S1497X(44?45?); |
|  | 2412 | **5** | Same | AML |  |  |  | DNMt3A/23/R882C(43)  ROBO2/13/R640H(43)  TET2/10/S1497X(40)  **CEBPA/1/A265fs(37)CEBPA/1/K90fs(52)**  EZH2/17/S652(100) |
| 1. F/62 | 1018 | **3** | 46，X,der（X) ，der(14） | RAEB2 | 2.5（7.0） | **1.5** | **3** | DNMT3A/8/R320X（48）  EZH2/12/R497Q（40）  TET2/6/R1214W（48） |
|  | 1061 | **5** | Same | AML |  |  |  | DNMT3A/8/R320X（29）  EZH2/12/R497Q（46）  TET2/6/R1214W（46）  BCOR/9R1341W(51)  **CEBPA/1/A295V(39)** |
| 11.M/59 | 843 | **2** | 47,XY,+der(1)del(1)(p21) | RCMD | 1.0（5.0） | **2.5** | **5** | SRSF2/1/P95R（38）  TET2/5/E1178fs（43）; |
|  | 962 | **3** | Same | AML |  |  |  | SRSF2/1/P95R（41）  TET2/5/E1178fs（40）  **TP53/5/C135X(31)** |
| 12.M/68 | 1831 | **3** | Complex | RAEB1 | 2.0（8.5）） | **13** | **13** | ANKRD11/9/A305T（46）  DNMT3A/20/G796fs(25)  **TP53/4/W91*** |
|  | 2322 | **6** | **complex** | AML |  |  |  | ANKRD11/9/A305T（46）  DNMT3A/20/G796fs(25)  **TP53/4/W91*（30）**  KIF20B/5/V137L(21)  STAG2/11/L360V(33)  **TP53/5/R158L(31)** |

**Note:** boldfaced characters in the “gene mutation” column presented the presumptive last events mutations met the criteria described in text
